# Studies with Azinylacetonitriles: 2-Pyridylacetonitrile as a Precursor to Functionally Substituted Pyridines

**DOI:** 10.3390/molecules14114406

**Published:** 2009-11-03

**Authors:** Mariam Abdullah Al-Sheikh, Mohamed Hilmy Elnagdi

**Affiliations:** 1 Department of Chemistry, Girls College of Education, Jeddah, P. O. Box 138016, Jeddah 21323, Kingdom of Saudi Arabia; 2 Department of Chemistry, Faculty of Science, Kuwait University, Kuwait

**Keywords:** 2-pyridylacetonitrile, enaminonitrile, arylhydrazones

## Abstract

2-Pyridylacetonitrile (**1**) couples with aromatic diazonium salts to yield arylhydrazones **2a-c**, that were shown to exist in the *syn*-form **2** rather than the *anti*-form **4**. Compounds **2a,c** reacted with hydroxylamine in refluxing DMF to yield the interesting 1,2,3-triazolylpyridines **6**. Attempts to cyclize **2** to give the corresponding fused pyrazolopyridines **9** failed. On the other hand, compound **1** condensed with dimethylformamide dimethyl acetal to yield enaminonitrile **10** that could be converted into pyrazolylpyridine **11**.

## Introduction

It is well accepted that the methylene moieties in heteroaromatic substituted acetonitriles are reactive toward electrophiles under mild conditions [1,2,3]. This reactivity has been utilized for synthesis of a variety of functionally substituted azoles [4,5] and condensed azoles [6,7]. However, little has been reported on the utility of azinylacetonitriles for synthesis of functionally substituted azines [8].

## Results and Discussion

**Figure 1:**
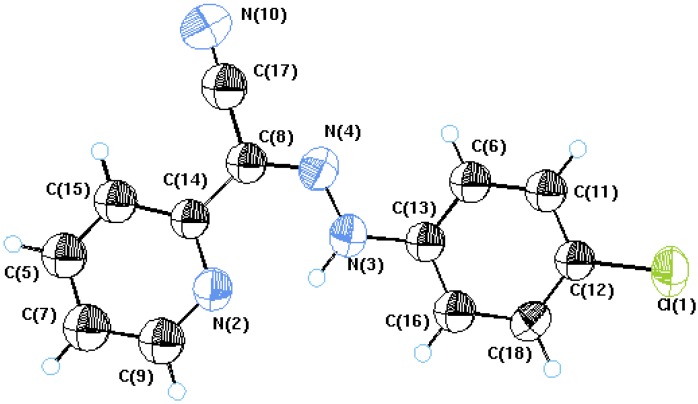
X-ray crystal structure of compound **2a.**

In conjunction with our interest in using aromatic and heteroaromatic substituted acetonitriles as precursors for the synthesis of heteroaromatics, we report herein the reactivity of 2-pyridylacetonitrile (**1**) as a good precursor to several azolylpyridines. Thus, compound **1** coupled with aromatic diazonium salts to yield arylhydrazones **2a-c**. Although a mixture of the two geometrical isomers was expected based on an earlier report [9], the existence of only the *syn*-structure **2** could be established, at least in the solid state, based on X-ray crystal structure determination [10] (cf. Figure 1 and Table 1 for bond angles and bond lengths). 

**Scheme 1:**
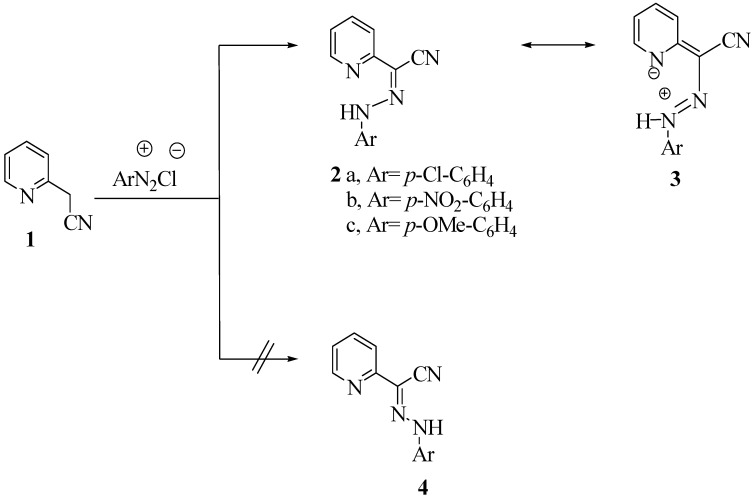
Syntheses of hydrazones.

**Table 1:** Crystal data and structure refinement for compound **2a**.

**Parameter**		**2a**	
Empirical Formula		C_13_H_9_N_4_Cl	
Formula weight		256.696	
Crystal System		Monoclinic	
Space group		P21/c	
Unit cell parameters			
a [Å]		8.4969(3)	
b [Å]		13.5879(6)	
c [Å]		12.8704(6)	
alpha		90.00	
B^o^		12.(18) 10^10^	
^gamma^		90.00	
Unit cell volumeZ		1228.62(9)4	
Temperature (K)		298	
Radiation type		Mo *K*a	
Dx Mg/m^3^		1.388	
F(000)		528 loop	
Absorption coefficient (mm^-1^)		0.30	
Parameters		103	
R factor		0.061	
**Bond lengths**		**Bond lengths**	
N4 C8	1.320(4)	N3 N4	1.326(4)
N2 C9	1.326(5)	N3 C13	1.412(5)
N2 H3	1.906(3)	C6 C13	1.363(5)
C8 C17	1.449(5)	Cl 1 C12	1.742(3)
N2 C14	1.349(4)	N10 C17	1.143(4)
N4 H3	1.985(3)	C13 C16	1.377(4)
**Bond angles**		**Bond angles**	
N3 N4 C8	118.6(3)	N2 C14 C8	116.9(3)
C14 C8 C17	118.5(3)	N2 H3 N3	132.7(2)
N2 C9 C7	124.3(4)	N3 H3 N4	35.4(2)
C8C17 N10	179.3(4)	C8 C14C15	122.1(3)
N3C13 C6	122.2(3)	N4 C8 C14	130.4(3)
N4 N3 C13	118.4(3)	N3 C13C16	117.6(3)

Inspection of Table 1 indicates that the acetonitrile N4-C8-C14 bond angle is larger than a typical sp^2^ bond angle (120^o^), perhaps to reduce the steric interaction between hydrogen NH, and the pyridyl ring N3-N4 bond length is more like a double bond. We assume that nitrogen lone pair is delocalized at the ring nitrogen and that electrostatic attraction between the positively charged hydrazone moiety and the negatively charged ring nitrogen holds the molecule in the *syn*-form. It is thus concluded that charge separation in **2** contributes significantly to the actual structure [10,11,12].

**Scheme 2:**
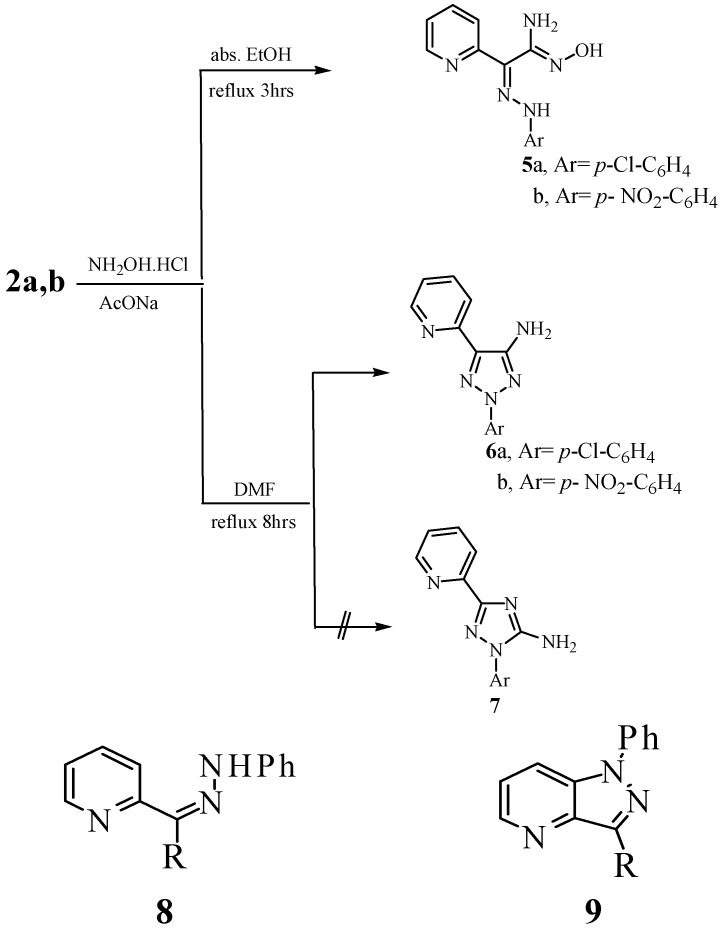
Syntheses of 1,2,3-triazoles.

**Scheme 3:**
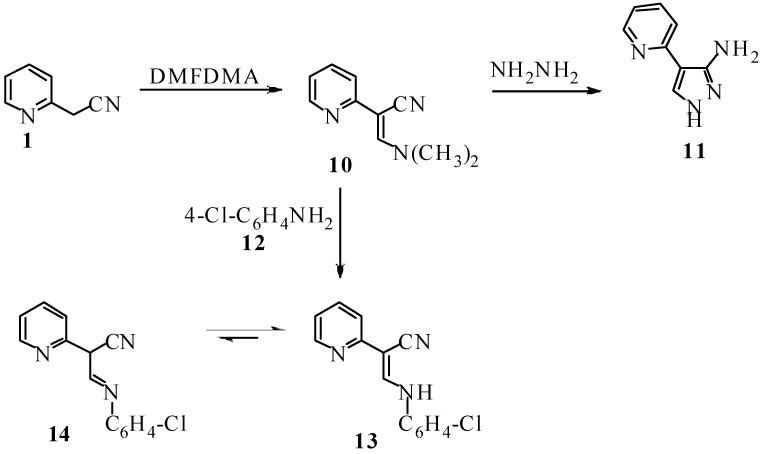
Reactions of the enaminonitrile **10**.

Compounds **2a,b** reacted with hydroxylamine hydrochloride in refluxing DMF and in the presence of sodium acetate to yield the products of addition and water elimination which can thus be formulated as 1,2,3-triazoles **6a,b **or their isomeric structures 1,2,4-triazoles **7**, and are assumed to be formed *via* the intermediately formed amidoximes **5a,b** that could be isolated (Scheme 2). NOE difference spectra enabled the assignment of structure **6** for the products as irradiation of the NH_2_ protons at δ= 6.3 ppm did not enhance the aryl protons. If the reaction product were **7** enhancement of these aryl protons should have been observed. The behavior of **2** towards hydroxylamine is thus similar to that of other hydrazononitriles and differs from that of 2-*p*-nitrophenyl-2-arylhydrazonoacetonitrile where rearrangement preceded cyclization affording 1,2,4-triazoles **7**. Although 2-arylhydrazono-2-acetylpyridine **8** has been recently reported to afford **9** when heating in dichlorobenzene at 190 °C, in our hands, compounds **2a-c** have been recovered unaffected under these conditions [13]. It seems that replacing a methyl by a cyano group affects the HOMO-LUMO energy of the cyclised 6π electron system as this cyclization is believed to proceed by a pericyclic rule. Next, compound **1** was reacted with DMFDMA to yield the corresponding enaminonitrile **10**, for which exact stereochemistry could not be established. Reacting **10** with hydrazine hydrate afforded aminopyrazole derivative **11,** in good yield (cf. Scheme 3). The ^1^H-NMR of compound **11** revealed three singlets at δ = 5.60, 7.90 and 11.76 ppm for the exocyclic NH_2_, pyrazole CH and pyrazole NH protons, respectively.

Compound **10** reacted with *p*-chloroaniline **12** to give compound **13**. Structure **14** was excluded based on ^1^H-NMR and ^13^C-NMR that revealed the absence of signals for a sp^3^ carbon or protons linked to such a carbon (Scheme 3). It can thus be concluded that while compound **1** is a versatile precursor to azolylpyridines, conversion of compound **2** to pyrazolo[4,3-*b*]pyridine *via* a route similar to that reported for converting **8** into **9** could not be effected, at least under the conditions reported in the published work [13].

## Experimental

### General

All melting points were measured on Gallenkamp electrothermal melting point apparatus and are uncorrected. Microwave synthesis were carried out in SJO390W microwave oven. IR spectra were recorded as KBr pellets on a Pye Unicam SP 3-300 spectrophotometer. ^1^H-NMR spectra were recorded in deuterated dimethylsulfoxide (DMSO-*d6*) at 300 MHz on a Varian Gemini NMR spectrometer using tetramethylsilane (TMS) as an internal reference and results are expressed as δ values. Mass spectra were performed on a Shimadzu GCMS-QP 1000 Ex mass spectrometer at 70 eV. Elemental analyses were performed by the Microanalytical Center at Cairo University. The crystal structure was determined by the X-ray unit at the National Research Center, Dokki, Cairo.

### General procedure for the synthesis of arylhydrazones ***2a-c***

A cold solution of aryldiazonium salt (10 mmol), prepared by adding a solution of sodium nitrite (10 mmol in 10 mL of water) to a cold solution of arylamine hydrochloride (10 mmol of arylamine in 6 mL of HCl) with stirring, was added to a cold solution of 2-pyridylacetonitrile (**1**, 10 mmol) in ethanol (50 mL) containing sodium acetate trihydrate (10 mmol). The mixture was then stirred at room temperature for 1 h and the resulting solid product was collected by filtration, washed well with water, dried and recrystallized from ethanol.

*[(4-Chlorophenyl)hydrazono]pyridine-2-yl-acetonitrile* (**2a**): Orange crystals (92%); mp.147-148 °C; IR (cm^-1^): 3258 (NH), 2212 (CN), ^1^H-NMR: δ = 7.50-8.10 (m, 4H, pyr-H), 8.58 (d, 2H, *J =* 7.2 Hz, Ar-H), 8.70 (d, 2H, *J =* 7.2 Hz, Ar-H), 15.03 (s, 1H, NH); Anal. Calcd. for C_13_H_9_ClN_4_ (256.69): C, 60.83; H, 3.53; N, 21.83. Found: C, 60.64; H, 3.60; N, 21.64. MS (EI): *m/z* (%) = 255 (M^+^-1).

*[(4-Nitrophenyl)hydrazono]pyridine-2-yl-acetonitrile* (**2b**): Orange crystals (90%); mp. 217-219 °C; IR (cm^-1^): 3241 (NH), 2218 (CN); ^1^H-NMR: δ = 7.40-8.20 (m, 4H, pyr-H), 8.63 (d, 2H, *J =* 7.3 Hz, Ar-H), 8.65 (d, 2H, *J =* 7.3 Hz, Ar-H), 15.25 (s, 1H, NH); Anal. Calcd. for C_13_H_9_N_5_O_2_ (267.24): C, 58.43; H, 3.39; N, 26.21. Found: C, 58.27; H, 3.29; N, 25.91. MS (EI): *m/z* (%) = 267 (M^+^).

*[(4-Methoxyphenyl)hydrazono]pyridine-2-yl-acetonitrile* (**2c**): Yellow crystals (81%); mp.181-182 °C; IR (cm^-1^): 3250 (NH), 2216 (CN); ^1^H-NMR: δ = 3.70 (s, 3H, CH_3_), 6.90-8.07 (m, 4H, pyr-H), 8.60 (d, 2H, *J =* 7.0 Hz, Ar-H), 8.70 (d, 2H, *J =* 7.0 Hz, Ar-H), 15.03 (s, 1H, NH);^ 13^C-NMR: δ = 156.1, 151.9, 150.0, 147.7, 139.1, 136.2, 123.2, 121.1, 119.1 (CN), 116.8, 114.8, 55.3 (OCH_3_); Anal. Calcd. for C_14_H_12_N_4_O (252.27): C, 66.65; H, 4.79; N, 22.21. Found: C, 66.72; H, 4.82; N, 22.28. MS (EI): *m/z* (%) = 252 (M^+^).

### General procedure for the synthesis of compounds ***5a,b***

To a mixture of arylhydrazononitriles **2a,b** (10 mmol) and hydroxylamine hydrochloride (10 mmol) in absolute ethanol (20 mL), anhydrous sodium acetate (2g) was added and the reaction mixture was then refluxed for 3 hrs. After cooling to room temperature, the mixture was poured into water and the resulting precipitate collected by filtration, washed with water, dried and recrystallized from ethanol.

*2-[(4-Chlorophenyl)hydrazono]-N-hydroxy-2-yl-acetamidine* (**5a**): Yellow crystals (80%); mp.164-165 °C; IR (cm^-1^): 3495 (OH), 3390, 3273 (NH_2_), 3185 (NH); ^1^H-NMR: δ = 5.60 (s, 2H, NH_2_), 7.20-8.0 (m, 4H, pyr-H), 8.50 (d, 2H, *J =* 7.1 Hz, Ar-H), 8.70 (d, 2H, *J =* 7.1 Hz, Ar-H), 10.17 (s, 1H, NH), 12.8 (s, 1H, OH); Anal. Calcd. for C_13_H_12_ClN_5_O (289.73): C, 53.89; H, 4.17; N, 24.17. Found: C, 53.79; H, 3.99; N, 24.20. MS (EI): m/z (%) = 288 (M^+^-1).

*2-[(4-Nitrophenyl)hydrazono]-N-hydroxy-2-yl-acetamidine* (**5b**): Orange crystals (80%); mp.284-285 °C; IR (cm^-1^): 3495 (OH), 3382, 3270 (NH_2_), 3179 (NH); ^1^H-NMR: δ = 5.20 (s, 2H, NH_2_), 7.40-8.0 (m, 4H, pyr-H), 8.30 (d, 2H, *J =* 7.0 Hz, Ar-H), 8.70 (d, 2H, *J =* 7.0 Hz, Ar-H), 8.90 (s, 1H, NH), 15.2 (s, 1H, OH); Anal. Calcd. for C_13_H_12_O_3_N_5_ (300.27): C, 52.0; H, 4.03; N, 27.99. Found: C, 52.10; H, 3.98; N, 28.02. MS (EI): *m/z* (%) = 300 (M^+^).

### General procedure for the synthesis of compounds ***6a,b***

To a mixture of arylhydrazononitriles **2a,b** (10 mmol) and hydroxylamine hydrochloride (10 mmol) in DMF (20 mL), anhydrous sodium acetate (2 g) was added. Then, the reaction mixture was refluxed for 8 hrs. The solvent was evaporated under vacuum and the crude product was collected by filtration, washed with ethanol, dried and recrystallized from ethanol/dioxane.

*2-(4-Chlorophenyl)-5-pyridin-2-yl-2H-* [1,2,3] *triazol-4-ylamine* (**6a**): Yellow crystals (80%); mp.167-169^o^C; IR (cm^-1^): 3303, 3155 (NH_2_); ^1^H-NMR: δ = 6.30 (s, 2H, NH_2_), 7.30-8.0 (m, 4H, pyr-H), 8.02 (d, 2H, *J =* 7.2 Hz, Ar-H), 8.6 (d, 2H, *J =* 7.2 Hz, Ar-H);^ 13^C-NMR: δ = 152.8, 150.7, 148.8, 137.9, 137.2, 132.1, 130.3, 129.4, 122.3, 119.7, 118.7; Anal. Calcd. for C_13_H_10_ClN_5_ (271.70): C, 57.47; H,3.71; N, 25.78. Found: C, 57.31; H, 3.68; N, 25.89. MS (EI): *m/z* (%) = 271 (M^+^).

*2-(4-Nitrophenyl)-5-pyridin-2-yl-2H-*[1,2,3] *triazol-4-ylamine* (**6b**): Orange crystals (79%); mp. 280 °C; IR (cm^-1^): 3336, 3278 (NH_2_); ^1^H-NMR: δ = 6.50 (s, 2H, NH_2_),7.30-8.11 (m, 4H, pyr-H), 8.30 (d, 2H*, J =* 7.3 Hz*,* Ar-H), 8.68 (d, 2H, *J =* 7.3 Hz, Ar-H);^ 13^C-NMR: δ =157.1, 150.0, 148.4, 137.7, 135.0, 132.1, 130.0, 129.0, 124.0, 122.0, 119.0, Anal. Calcd. for C_13_H_10_N_6_O_2_ (282.26): C, 55.32; H, 3.57; N, 29.77. Found: C, 55.20; H, 3.46; N, 29.69. MS (EI): *m/z* (%) = 282 (M^+^).

*Synthesis of 3-dimethylamino-2-pyridin-2-yl-acrylonitrile* (**10**): A mixture of compound **1** (10 mmol) and dimethylformamide dimethylacetal (DMFDMA) (10 mmol) was irradiated in a domestic microwave oven for 1 minute at 240 W. The mixture was left standing overnight and the resulting solid product was collected by filtration, washed with ethanol, dried and recrystallized from ethanol to give compouned **10** as brown crystals (80%), mp.116-118 °C; IR (cm^-1^): 2221 (CN); ^1^H-NMR: δ = 2.48 (s, 6H, 2CH_3_), 6.90-8.60 (m, 4H, pyr-H), 8.07 (s, H, olefinic CH); Anal. Calcd. for C_10_H_11_N_3_ (173.21): C, 69.34; H, 6.40; N, 24.26. Found: C, 69.41; H, 6.41; N, 24.17. MS (EI): *m/z* (%) = 173 (M^+^).

*Synthesis of 4-pyridin-2-yl-2H-pyrazol-3-yl-amine* (**11**): **A** mixture of compound **10** (10 mmol) and hydrazine hydrate (80%, 10 mmol) was irradiated in a domestic microwave oven for 2 minutes. The resulting solid product was collected by filtration, washed with ethanol, dride and recrystallized from ethanol to give compound **11** as brown crystals (75%); mp. 120-121 °C; ^1^H-NMR: δ = 5.60 (s, 2H, NH_2_), 6.90-8.40 (m, 4H, pyr-H), 7.90 (s, 1H, pyrazole H-5), 11.76 (s, 1H, NH);^ 13^C-NMR: δ = 155.1, 151.1, 148.8, 136.8, 133.4, 119.0, 118.7, 103.0; Anal. Calcd. for C_8_H_8_N_4_ (160.20): C, 59.99; H, 5.03; N, 34.98. Found: C, 60.01; H, 4.98; N, 34.87. MS (EI): m/z (%) = 160 (M^+^).

*Synthesis of 3-(4-chlorophenylamino)-2-pyridin-2-ylacrylonitrile* (**13**): To a mixture of *p*-chloroaniline **12** (10 mmol) and compound **10** (10 mmol), a drop of AcOH was added, then the mixture was irradiated in a domestic microwave oven for 2 minutes at 280 W. The resulting solid product was collected by filtration, washed with ethanol, dried and recrystallized from ethanol to give compound **13** as colourless crystals (86%); mp. 179-180 °C; IR (cm^-1^): 3387 (NH), 2202 (CN); ^1^H-NMR: δ = 7.1-8.2 (m, 4H, pyr-H), 8.40 (d, 2H, *J =* 7.2 Hz, Ar-H), 8.50 (s, H, olefinic CH), 8.60 (d, 2H, *J =* 7.2 Hz, Ar-H), 12.60 (brs, 1H, NH);^ 13^C-NMR: δ = 156.0, 150.0, 149.0, 144.0, 137.0, 130.0, 123.0, 122.0, 121.0, 119.0 (CN), 116.0, 103.0; Anal. Calcd. for C_14_H_10_ClN_3_ (255.7): C, 65.76; H, 3.94; N,16.43. Found: C, 65.59; H, 3.99; N, 16.52. MS (EI): *m/z* (%) = 254 (M^+^-1).

## Conclusions

In conclusion, a new simple approach to 2,5-disubstituted-1,2,3-triazole-5-amines from 2-aryl-hydrazononitriles has been achieved.
